# Sodium metabisulfite-induced changes on testes, spermatogenesis and epididymal morphometric values in adult rats

**Published:** 2015-12

**Authors:** Shahnaz Shekarforoush, Zahra Ebrahimi, Maryam Hoseini

**Affiliations:** *Department of Physiology, Islamic Azad University, Arsanjan Branch, Fars, Iran.*

**Keywords:** *Sodium metabisulfite*, *Sperm*, *Testosterone*, *Epididymis*

## Abstract

**Background:**

Sulphites are widely used as a preservative and antioxidant additives in the food and pharmaceutical industries. Many types of biological and toxicological effects of sulphites in multiple organs of mammals have been shown in previous studies.

**Objective:**

The aim of this study was to investigate the effects of sodium metabisulfite (SMB) on testicular function and morphometric values of epididymis in adult male Wistar rats.

**Materials and Methods:**

A total of 32 rats were randomly divided into four groups. The experimental groups received SMB at doses of 10 mg/kg (S10), 100mg/kg (S100), and 260 mg/kg (S260) while an equal volume of normal saline was administered to the control group via gavage. The rats were anaesthetized after 28 days and the left testis with the head of epididimis was excised following abdominal incision for histological observation using hematoxylin and eosin staining. Serum samples were collected for assay of testosterone level. The initial epididymis was analyzed for motility, morphology, and the number of sperms.

**Result:**

The results of this study showed that normal morphology, count, and motility of sperms and testosterone level were decreased in the SMB treated groups. In comparison with the control group, SMB resulted in a lower total number of spermatogonia, primary spermatocyte, spermatids, and Leydig cells.

**Conclusion:**

It is suggested that SMB decreases the sperm production and has the potential to affect the fertility adversely in male rats.

## Introduction

Sulfite salts and sulphur dioxide are added as preservatives to a variety of foods, such as biscuits, chocolate, jam, and in many alcoholic beverages such as beer, wine, etc. and in medications such as parenteral amino acid solutions (1). Endogenous sulfites are generated in vivo by the catabolism of sulfur-containing amino acids, cysteine and methionine (2).

Sulfite salts including sodium metabisulfite (SMB), potassium metabisulfite, sodium bisulfite, potassium sulfite, and sodium sulfite have all been termed as “sulfating agents” because they release sulphur dioxide. Federation of American Societies for Experimental Biology estimates a "no-observed-adverse effect level" of 30-100 mg sulfur dioxide in humans (3). Some studies have indicated that sulfites produce toxic effects on the reproductive system, the respiratory system (4), the nervous system (5) and can also induce allergic reactions (1). In earlier studies, it has been reported that sodium metabisulfite and sulfur dioxide cause an increase in the lipid peroxidation (2, 6).

After entering the body via ingestion, inhalation, or injection, sulfites are metabolized by sulfite oxidase, an enzyme located in the inter-membranous space of the mitochondria to sulfate (3). Different tissues exhibit different sulfite oxidase activities. Testis has a very low sulfite oxidase activity, suggesting that the testes are highly sensitive to sulfite toxicity (7).

Testis, the organ that produces sperm and androgens, is sensitive to a variety of stressors and agents that induce germ cell apoptosis (1). Understanding these agents and studying the effects of these factors on the male reproductive function is essential in the maintenance of male fertility. It is known that sulphur dioxide is a toxin to the reproductive system and can lead to high level of oxidative stress in the testicles of male mice (4). 

Sulphur dioxide results in decreased protein levels and increased lactate dehydrogenase activities in the testis of male rats (8).

Regarding the effects of SMB on the male reproductive system, scientific data are limited. The aim of the present study was to investigate the effects of sodium metabisulfit ingestion on testicular function and epididymal morphometric values in adult rats.

## Materials and methods

This is an experimental study performed in animal house of Shiraz University in 2014. In total 32 male Wistar rats weighing 220-250 g were obtained from animal house of Shiraz University of Medical Sciences, Iran. They were maintained at 12h light-dark cycles and temperature of 23˚C ± 2˚C with free access to food and water. All experimental protocols were performed in accordance with the Ethics Committee of Shiraz University of Medical Sciences.

**Drugs preparation**

SMB was obtained from Sigma (EC No: 231-673-0, CAS. No: 7681-57-4). SMB was dissolved in distilled water. With reference to the reported theoretical yield of 67.39% Sulfur dioxide (SO2) from Na2S2O5 (3), the given dose of 10, 100 and 260 mg SMB/kg/day was equivalent to 7, 67 and 175 mg SO2. 

**Experimental design**

The rats were randomly divided into four groups of 8 animals each: 1) control group, which received distilled water, 2) S10 group, 3) S100 group, 4) S260 group, to which 10, 100 and 260 mg/kg b.w. of SMB was administered orally using feeding needle once a day for 28 consecutive days, respectively.

One day after the last administration, the rats were anesthetized using diethyl ether and after laparotomy blood samples were collected by cardiac puncture. 

Thereafter, the left testis and epididymis in 6 animals per group were removed and then the tissues were immediately fixed in 10% formaldehyde in buffered solution for 72 hours for histological studies.

**Histo-morphometric evaluation**

The tissues were processed through graded alcohols, cleared in xylene, and embedded in paraffin. Sections of 5 μm were cut and stained with hematoxylin and eosin. All the slides were examined under a light microscope (Nikon, H-108N-Japan). Ten fields chosen randomly from each histological section were measured for estimation of the total number of Leydig cells, sertoli cells, spermatogonia, spermatocytes, and round spermatids (8). 

The measurements of the epididymal tubular diameter and epithelial height were performed at 100x magnification using an ocular micrometer after calibration with a stage micrometer. The best choice of magnification for these measurements is 100× that is most commonly used (9).

**Serum testosterone measurements**

After centrifugation at 3500 rpm for 20 minutes, serum samples were separated, frozen and stored at −20˚C until further analyses. Concentration of testosterone was determined by ELISA assay kit (DRG Instruments GmbH, Germany). The sensitivity of hormone detection was 0.05 ng/ml. All the samples were analyzed in a single assay to avoid inter-assay errors. 

**Sperm analysis**

The samples were obtained from the cauda epididymis (1.0 cm) of all animals and placed in a plate containing 5 cc of normal saline at 37^º^C for 5 min. A drop of solution was transferred in a warm Neubauer hemocytometer (Merk Company, Germany). The sperm concentration, motility, and morphology were estimated in 10 separate randomly selected fields through a light microscope. Sperm count was expressed as million per ml by multiplying the mean numbers of sperms by a factor of one million.

To analyze sperm morphology, the sperm smears were left to dry for 5 min, fixed with methanol, stained with eosin and evaluated in 100 sperm per animal (100×) (10). Spermatozoa were morphologically classified as normal or abnormal. The abnormality was classified based on having head and tail defects. Sperm motility was classified as an immotile and motile with and without progressive movement. The percentage of motility was determined by counting both motile and immotile sperm and calculated from the mean numbers of sperms for all fields counted by a factor of one hundred/ total number of sperms. 

**Statistical analysis**

All data were expressed as Mean ± SEM or percentage. Statistical analysis was performed with SPSS 16, using with one-way ANOVA and Tukey post hoc. P<0.05 was 

considered as statistically significant. 

## Results

**Effect of SMB on testosterone**

**Figure 1:**
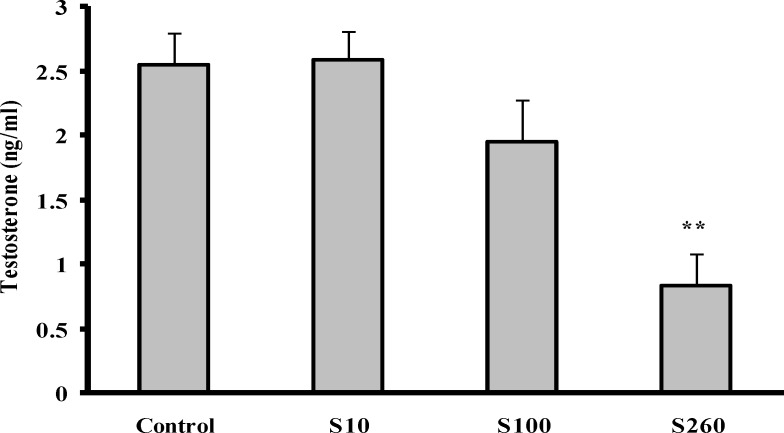
Effect of different concentrations of sodium metabisulfite on testosterone. (n=6, ** p< 0.01 difference vs. control group). S10, administration of 10 mg/kg/day SMB, S100, administration of 100 mg/kg/day SMB, S260, administration of 260 mg/ kg/day SMB

The results of this study revealed that the serum level of testosterone (ng/ml) in the S260 group decreased significantly in comparison with the control group (p= 0.001) (Figure 1).

**Testicular histology and epididymal morphometric analysis**

The number of different cells of the germinal epithelium and morphometric analysis of the epididymis of rats is shown in Table I. According to the results, the total number of spermatogonia, primary spermatocyte, spermatids and Leydig cells in animals treated with the SMB significantly decreased in comparison with the control group. The total number of sertoli cells did not show any significant difference in any of the groups. The treated groups showed a significant decrease in the mean diameter of the epididymal tubules and mean height of the epithelial cell compared to the controls.

**Table I:** The number of the different cells of germinal epithelium and morphometric analysis of epididymis of the control and sodium metabisulfite treated groups

	**Control**	**S10**	**S100**	**S260**
Spermatogonia	85.38 ±1.98	82.97 ±2.12	57.50 ±0.72**	60.25 ±2.96**
Spermatocyte	119.91 ±7.84	110.95 ±4.58	73.16 ±7.15**	75.16 ±2.51**
Spermatid	187.05 ±6.54	186.66 ±6.53	118.88 ±9.27**	115.80 ±10.43**
Sertoli cells	20.76 ±1.62	25.83 ±3.38	20.91 ±1.76	21.38 ±1.20
Leydig cells	40.02 ±1.47	38.58 ±1.34	29.64 ±1.82**	26.97 ±2.19**
**Epididymal morphometry **				
Tubular diameter (µm) Epithelium height (µm)	186.83 ±11.79 10.20 ±0.60	184.03 ±12.57 9.99 ±0.54	159.04 ±14.72 6.66 ±0.52*	128.32 ±14.33* 5.25 ±0.51***

Each value indicates the mean ± SEM. S10, administration of 10 mg/kg/day SMB, S100, administration of 100 mg/kg/day SMB; S260, administration of 260 mg/kg/day SMB.

* p<0.05,

** p<0.01,

*** p<0.001 vs. the control group.

**Sperm Analysis**

The sperm count was decreased in the S260 group (about 42% decline). The data revealed that normal morphology sperm percentage was reduced significantly in the S100 and S260 groups. The immotile sperm were significantly increased in the S260 group in comparison with the control group (p<0.001), as shown in table II.

**Table II:** Sperm analysis data of the control and sodium metabisulfite treated groups

	**Control**	**S10**	**S100**	**S260**
Sperm count (10^6^/ml)	74.5 ±11.2	77.3 ±11.9	59.5 ±7.9	43.6 ±7.1
Normal morphology (%)	68.8 ± 3.1	62.1 ± 3.1	42.2 ±1.4***	23.2 ±3.5***
Motility				
Progressive (%)	65.9 ±1.8	67.9 ±2.2	63.2 ±3.1	58.6 ±3.7
Nonprogressive (%)	18 ±1.5	17.6 ±2.8	14.4 ±1.9	9.2 ±1.5*
Immotile (%)	14 ±1.9	15.7 ±1.8	21.6 ±2.6	31.6 ±2.9***

Each value indicates the mean ± SEM. S10, administration of 10 mg/kg/day SMB; S100, administration of 100 mg/kg/day SMB; S260, administration of 260 mg/kg/day SMB.

* p<0.05,

*** p<0.001 vs. the control group.

## Discussion

The main findings of the present study showed that SMB led to: 1) decreased total number of spermatogonia, primary spermatocyte, spermatid and Leydig cells, 2) decreased sperm count, motility and increased sperm abnormality, 3) decreased epididymal tubule diameter and its epithelium height, and 4) decreased the serum levels of testosterone.

It is estimated that daily sulfite intake from foods and beverages is 180–200 mg/Kg body weight, which is not in agreement with acceptable daily intake level of 0.7 mg/kg body weight (11). Several studies have shown that ingestion of SMB at a dose of 520mg/kg/day induced oxidative damage in the rats’ testes, liver, and kidney (1, 12). The doses of 10,100, and 260 mg/kg/day sulfite were selected from previous studies (6, 13), designed to represent human exposure to different levels of sulfite, which occurred through the consumption of certain foods and drugs.

There is some evidence to suggest that sulfites have toxic effects on several organs and tissues. The mechanism by which accumulation of sulfites changes the cellular function has not been fully elucidated. It seems that sulfite toxicity increased by producing sulfite radical capable to react with DNA, lipids, and proteins (14). SMB induces lipid peroxidation as a dose-dependent process in the rat’s gastric tissue (13) to indicate that lipid peroxidation could play an important role in sulfite toxicity. This was supported by the studies of Derin *et al* (11) and Izgüt-Uysal *et al* (15), demonstrating that some antioxidants such as vitamin E can at least in part prevent sulfites induced oxidative damage in various tissues.

The activity of sulfite oxidas is low in the brain, spleen, and testis (16) compared with other tissues, suggesting that the testes are highly sensitive to sulfite toxicity. SO2 effects on male reproduction have recently been demonstrated in epidemiological investigations (17). Alteration in testis enzyme activities through inducing the production of free radicals by sulfate and sulfite are one of the pathways of decreased semen quality (18). Testosterone is a critical germ cell survival factor because its removal induces germ cell apoptosis (19) and it is essential for maintaining spermatogenesis and male fertility (20). 

The present study showed that SMB reduces testosterone, a reduction of 68%. The mammalian epididymis is no longer regarded as a mere conduit pipe for spermatozoa from the testis to out, but it equally serves a critical function in preparing the male germ cells for fertilization. The epididymis to maintain its structure and function depends on androgens (21). The morphometric alteration in epididymis seen in the SMB treated groups might be due to testosterone decline. 

## Conclusion

According to the present results, SMB caused a reduction in the testosterone level, improper spermatogenesis and an alteration in epididymal morphometry in rats. 

The effect of SMB exposure on spermatogenesis in rats has raised concerns for potential effects on humans. Therefore, a research on materials that can reduce its side effects is necessary.

## Note

***This article extracted from M.Sc. thesis (Zahra Ebrahimi and Maryam Hoseini)***
